# Structural and Metabolic Remodeling of Skeletal Muscle in Heart Failure with Reduced Ejection Fraction: A Review: Beyond the Failing Heart

**DOI:** 10.3390/ijms27062886

**Published:** 2026-03-23

**Authors:** Mamata Chaudhari, Jamila Makhloufi, Benjamin Doelling, Raveena Kataria, Aruni Bhatnagar, Dinesh Kalra, Shahid Pervez Baba

**Affiliations:** 1 Center for Cardiometabolic Science, Louisville, KY 40202, USA; mamata.chaudhari@louisville.edu (M.C.); jamila.makhloufi@louisville.edu (J.M.); benjamin.doelling@louisville.edu (B.D.); raveenaravinder.kataria@louisville.edu (R.K.); aruni@louisville.edu (A.B.); 2 Christina Lee Brown Envirome Institute, Louisville, KY 40202, USA; 3 Division of Cardiovascular Medicine, University of Louisville School of Medicine, Louisville, KY 40202, USA

**Keywords:** carnosine, exercise intolerance, heart failure, glucose, mitochondria, skeletal muscle

## Abstract

Heart failure (HF) with reduced ejection fraction is a systemic disorder that extends beyond cardiac dysfunction and involves peripheral organs, particularly skeletal muscle. Exercise intolerance and fatigue are the hallmark manifestations of HF that strongly predict morbidity and mortality. Accumulating evidence suggests that intrinsic skeletal muscle abnormalities are key contributors to exercise intolerance in HF. In HF, skeletal muscle undergoes metabolic remodeling characterized by shifts in fiber type composition, mitochondrial dysfunction, and increased oxidative stress. Mitochondrial dysfunction, characterized by decreased mitochondrial density, impaired biogenesis, and reduced respiratory capacity, further compromises skeletal muscle performance. These alterations impair adenosine triphosphate (ATP) generation via oxidative phosphorylation, forcing reliance on less efficient anaerobic glycolysis. The resulting metabolic shift exacerbates early lactate accumulation, muscle fatigue, and diminished exercise capacity. In parallel, an increase in oxidative and carbonyl stress, along with a decrease in antioxidant defenses as well as derangements in pathways that remove toxic lipid peroxidation, heightens oxidative and carbonyl stress perpetuating injury and establishing a vicious cycle of progressive muscle dysfunction. Thus, metabolic remodeling in skeletal muscle represents a central determinant of exercise intolerance in HF. While exercise training remains the most effective strategy to restore skeletal muscle health and exercise tolerance, emerging therapies offer novel avenues for intervention. Future research should focus on elucidating the molecular mechanisms underlying skeletal muscle dysfunction and developing therapies that restore metabolic integrity and functional capacity in HF.

## 1. Introduction

Heart failure (HF) with reduced ejection fraction is a multifaceted clinical syndrome characterized by the heart’s inability to generate adequate cardiac output to meet the body’s metabolic demands. Among its systemic manifestations, exercise intolerance is a cardinal hallmark that profoundly diminishes quality of life and serves as a predictor of adverse outcomes. Traditionally, exercise intolerance in HF patients was primarily viewed through the lens of central hemodynamic impairment. However, cardiotonic agents intended to improve symptoms and exercise intolerance in these patients failed to improve exercise capacity despite improving hemodynamics [1,2]. This observation helped shift the focus toward peripheral factors.

Accumulating evidence demonstrates that skeletal muscle dysfunction and altered metabolism play a central role to the symptomatology of exercise intolerance in HF. Wilson et al. first reported this concept by showing that, during dobutamine infusion, HF patients exhibited increased cardiac output and leg blood flow during exercise, yet displayed no improvements in maximal exercise duration, peak systemic VO_2_, or lactate kinetics, indicating that intrinsic skeletal muscle defects substantially contribute to exertional fatigue in HF [3].

The purpose of this review is to summarize the structural, metabolic, and molecular alterations in skeletal muscle in HF with reduced ejection fraction. Furthermore, we will discuss emerging therapeutic interventions aimed at restoring skeletal muscle function and improving exercise tolerance in this patient population. Although, approximately half of all HF patients have preserved ejection fraction (HFpEF) and exhibit impaired exercise performance. Addressing the complex pathophysiological heterogeneity and the contribution of individual components to exercise intolerance in this patient population is beyond the scope of this review. Therefore, this review focuses on the contribution of skeletal muscle pathology to exercise intolerance in HF with reduced fraction.

## 2. Alterations in Skeletal Muscle Structure and Fiber Type During Heart Failure

Myocytes are the basic cellular units of skeletal muscle and contain a highly organized array of contractile proteins within sarcomeres, the fundamental functional units of striated muscle. Each sarcomere spans a few micrometers from Z-line to Z-line and consists of thick myosin and thin actin arranged in a repeating pattern that determines the contractile properties. The thin filament contains the regulatory proteins tropomyosin (Tm) and troponin (Tn), which mediate Ca^2+^-dependent regulation of contraction. When Ca^2+^ binds to Tn, Tm repositions, exposing myosin binding sites on actin and enabling cross-bridge cycling. Titin, a large elastic protein extending from the Z-line to the M-line, maintains sarcomere integrity and contributes to passive stiffness. In the relaxed state, myosin heads adopt a low-energy “super relaxed” conformation stabilized by Mg-ATP and low cytosolic Ca^2+^. Activation initiates asynchronous cross-bridging interactions between myosin heads and actin filaments, driven by ATP hydrolysis and regulated by Ca^2+^ binding [4].

Alterations in contractile protein composition and kinetics contribute significantly to the reduced skeletal muscle strength in HF. Reduced force production in HF is partly due to loss of myosin heavy chain (MHC) content and diminished myofibrillar density. In rat models of coronary artery ligation, maximal specific force, MHC abundance, and Ca^2+^ sensitivity of contraction is markedly reduced in diaphragm fibers [5]. Similarly, in HF patients, single myofibril studies reveal decreased MHC content, fewer cross-bridges, and lower maximal Ca^2+^-activated tension [6,7]. Beyond quantitative deficits, intrinsic alteration in myosin kinetics exacerbates functional decline. In vitro motility assays using purified myosin isolated from HF skeletal muscle show slowed cross-bridge cycling characterized by prolonged actomyosin attachment times, reduced Ca^2+^ sensitivity, and altered viscoelastic properties, indicating intrinsic molecular defects in contractile performance [8,9,10].

**Figure 1:**
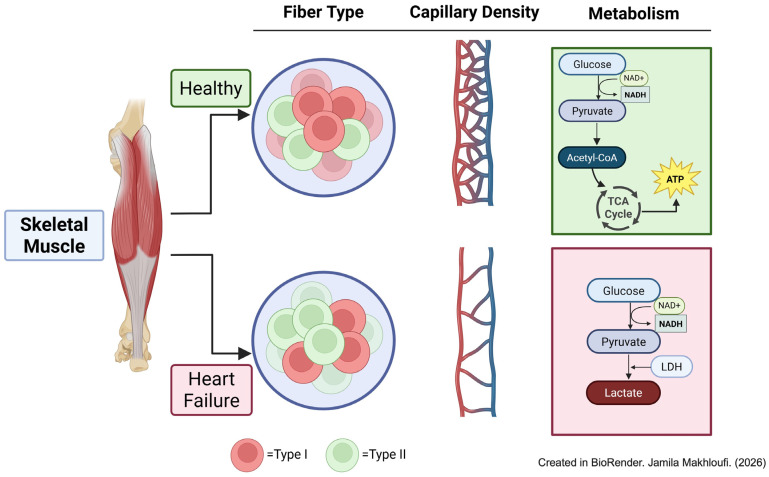
Skeletal muscle fiber composition, perfusion, and metabolic remodeling in heart failure. Skeletal muscle undergoes remodeling during heart failure characterized by a shift toward glycolytic fibers, reduced capillary density and blood flow, and a transition from oxidative to predominantly anaerobic metabolism.

Skeletal muscle fibers are categorized into slow-twitch (Type I) and fast-twitch (Type II) based on their MHC isoforms. Fast-twitch fibers are further subdivided into IIa, IIx, and IIb. Type I and IIa fibers rely primarily on oxidative metabolism, whereas IIx and IIb rely on glycolytic metabolism [4]. Both human and animal studies demonstrate consistent remodeling of MHC composition in HF, profoundly affecting muscle function. In the rat coronary ligation model, expression of fast MHC isoforms (MHC IIx and IIb) increases in gastrocnemius and plantaris muscles, correlating with HF severity [11]. In dogs with chronic HF, type I fibers decrease while glycolytic type II fibers increase [12]. Mancini et al. reported increased type IIb fibers, selective atrophy of type II fiber, and appearance of type IIc fibers [13]. Histological studies reveal intramyocellular lipid accumulation endomysial fibrosis within type I fibers and type II fiber hypotrophy [14]. Lipkin et al. further described atrophy of both type I and II fibers, increased interstitial cellularity, lipid deposition, and acid phosphatase staining in HF biopsies [15]. This shift in the fiber type correlates with HF severity, higher NYHA functional class associated with increased MHCII expression and decreased MHCI abundance [16,17,18]. Collectively, these studies indicate maladaptive remodeling marked by loss of oxidative fibers, expansion of glycolytic fibers, and impaired contractile protein composition (Figure 1).

## 3. Skeletal Muscle Metabolism

### 3.1. Insulin Resistance and Exercise Intolerance

Insulin resistance is a common metabolic abnormality in HF and is strongly associated with reduced exercise capacity and adverse clinical outcomes [19,20]. Impaired insulin sensitivity correlates with skeletal muscle weakness and increased mortality risk [19]. In HF, skeletal muscle glucose uptake is reduced by ~20% compared with healthy individuals, and this impairment is consistently observed in both ischemic heart disease and idiopathic dilated cardiomyopathy. These observations suggest that systemic insulin resistance may arise secondary to HF rather than being directly related to ventricular dysfunction.

Although reduced skeletal muscle blood flow and abnormal sympathetic nervous system are present in HF, insulin resistance is primarily attributed to intrinsic metabolic and biochemical defects in the skeletal muscle. Normally, insulin signaling in skeletal muscle involves a cascade of phosphorylation events, leading to the activation of insulin receptor substrates (IRS), AKT phosphorylation, and the translocation of GLUT4 to the plasma membrane, facilitating glucose uptake. In HF, insulin signaling defects do not occur at the IRS-1/PI-3kinase/AKT phosphorylation levels. Instead, there is a reduction in GLUT4 transport protein in skeletal muscle [21]. Physiological hyperinsulinemia increases tyrosine phosphorylation of IRS and IRS-associated PI3-K; however, skeletal muscle glucose uptake in skeletal muscle remains unchanged [22]. Similarly, in mouse models of myocardial infarction (MI), protein levels and phosphorylation status of IRS-β, IRS-1, and PI-3K remain unchanged under both baseline and insulin-stimulated conditions, but serine phosphorylation of AKT and GLUT4 translocation are decreased in insulin-stimulated skeletal muscle [23]. Increased reactive oxygen species generated via NADPH oxidase have been reported to impair insulin signaling at the Akt level in HF skeletal muscle [23]. Recent findings also show that skeletal muscle-specific microRNA-133b, which targets the 3′-untranslated region (UTR) of KLF15- a transcription factor regulating GLUT4, is increased in the skeletal muscle of HF mice. Lowering miR-133b levels in skeletal myocytes improves glucose uptake and restores GLUT4 abundance in skeletal muscle [24].

Insulin resistance is additionally associated with reduced coronary flow reserve (CFR), a marker of coronary micro vessel formation [25], and improving insulin sensitivity via exercise training improves CFR [26]. However, it is not yet known whether improvements in CFR through exercise training directly translate into downstream enhancements in skeletal muscle metabolic function. Nonetheless, insulin resistance is intractably linked with skeletal muscle dysfunction and exercise intolerance.

### 3.2. Shift in Glucose Metabolism

In healthy individuals, skeletal muscle uses both glucose and fatty acids to produce ATP through oxidative phosphorylation; approximately 50% of absorbed muscle glucose is oxidized, 35% is stored, and 15% is released as lactate and pyruvate [27]. In HF skeletal muscle, however, metabolism shifts away from an efficient oxidative metabolism towards a less efficient glycolytic metabolism, unrelated to blood flow [28,29]. Phosphorus-31 (31-P) nuclear magnetic resonance studies demonstrate that, although baseline skeletal muscle pH is normal, HF patients experience a more rapid decline in pH during exercise and delayed metabolic recovery compared with normal subjects [30,31]. Lactate levels and lactate dehydrogenase activity are elevated, whereas oxidative enzyme activity, such as citrate synthetase, is decreased [32]. High-throughput RNA-Seq analysis of skeletal muscle from HF patients further confirms downregulation of lactate dehydrogenase C expression [33]. Collectively, these findings indicate a metabolic shift characterized by increased glycolytic and reduced oxidative enzyme activity in HF skeletal muscle.

## 4. Mitochondrial Defects in Skeletal Muscle

### 4.1. Ultrastructural Alteration in Mitochondria

Mitochondrial dysfunction occurs in skeletal muscle during the early, progressive, and late phases of heart failure. Severe heart failure patients exhibit reduced mitochondrial volume density, cristae surface density, and smaller mitochondria, indicating decreased oxidative capacity [34]. Mitochondria from the HF skeletal muscle present a hydropic degeneration, membrane disruption, and are smaller compared with the normal subjects [35]. Decreased mitochondrial volume correlates with peak exercise (VO_2_) and VO_2_ at anaerobic threshold [34]. Similarly, murine models of HF show reduced mitochondrial cristae density and increased mitochondrial damage, parallel with a decrease in exercise capacity and mitochondrial respiration in the skeletal muscle fibers [36].

### 4.2. Mitochondrial DNA and Biogenesis

Mitochondria contain their own DNA (mtDNA), encoding 13 proteins essential for mitochondrial respiration, packaged with nuclear encoded proteins, including ATPase family AAA domain-containing protein 3 (ATAD3), mitochondrial transcription factor A (TFAM), and POLG (DNA polymerase gamma, catalytic subunit), forming an mtDNA–protein complex. Among these, TFAM is critical for maintaining mitochondrial function. Although several studies in human and mouse models of heart failure report no changes in the mtDNA in the skeletal muscle; TFAM expression is significantly reduced in the angiotensin-induced heart failure models [37] and in rats subjected to coronary ligation [38]. Interestingly, while TFAM expression remains unaltered in skeletal muscle of human heart failure patients compared to healthy subjects, resistance exercise training increases TFAM expression, correlating with improvements in the muscle strength [39].

Deletion of *Tfam* in the skeletal muscle causes myopathy, accumulation of abnormal mitochondria, and reduced muscle forces [40], while TFAM overexpression protects against atrophy changes during disuse [41]. Recent evidence shows that sodium-glucose cotransporters (SGLT2) inhibitors promote mitochondrial biogenesis by upregulating TFAM expression [42]. Moreover, SGLT2-inhibitor therapy in heart failure patients improves peak VO_2_ consumption and oxygen uptake efficiency [43,44].

### 4.3. Mitochondrial Dynamics

Mitochondria undergo continuous remodeling through fusion and fission. Mitochondrial fusion allows two mitochondria to merge at the outer and inner membrane interfaces, a process mediated by three GTPases: mitofusin1 (MFN1), mitofusin2 (MFN2) on the outer membrane, and optic atrophy protein (OPA1) on the inner membrane. MFN1 and 2 belong to the dynamin-related proteins (DRP) superfamily, while OPA1 is the mammalian ortholog of Mgm1 [45,46,47]. Outer mitochondrial fusion is facilitated by MFN1 and MFN2, while inner membrane fusion is mediated by OPA1 and specific inner mitochondrial components, such as cardiolipin [47]. Mitochondrial fission, on the other hand, divides a mitochondrion into two smaller mitochondria, which is crucial for removing damaged organelles and prepares the mitochondria to be removed via mitophagy. Fission is primarily coordinated by dynamin-related protein, a large dynamin-like GTPase. Other proteins involved in the fission process include dynamin 2, human mitochondrial dynamics proteins 49 and 51, mitochondrial fission factor 1 protein (MFP1), and mitochondrial fission factor (MFF) [47,48,49,50]. In addition to the fusion and fission, mitochondrial quality control is maintained by mitochondria-specific autophagy termed mitophagy. Here, PTEN-induced putative kinase (PINK1), activates Parkin, an E3 ubiquitin ligase, which ubiquitinates outer mitochondrial membrane protein of damaged mitochondria. This ubiquitination promotes the recruitment of downstream autophagy adaptor proteins, such as P62 and Beclin 1, which interact with LC3 and facilitate the selective degradation of impaired mitochondria via autophagy [51,52,53,54,55].

In the gastrocnemius muscle of mice subjected to coronary artery ligation, the balance between mitochondrial fission and fusion is disrupted, with a lower ratio of OPA19 (fusion) and DRP1 (fission) protein levels. This imbalance is driven by reduced OPA1 protein content, suggesting increased mitochondrial fragmentation [56]. Interestingly, sex-specific divergences are observed in human heart failure patients. While transcript levels of MFN2 and DRP1 remain unchanged in the skeletal muscle of male HF patients, OPA1 expression is uniquely lower in female HF patients [57,58]. However, expression of F1S1, a regulator of mitochondrial fission, is unaffected, indicating a shift in equilibrium toward mitochondrial fragmentation and network disruption. OPA1 expression correlates with whole-body oxygen consumption (VO_2_ peak), suggesting that impaired mitochondrial fusion not only reduces mitochondrial capacity but also contributes to skeletal muscle dysfunction and worsens exercise tolerance.

Very few studies have directly examined the role of PINK1-Parkin pathway in regulating the skeletal muscle mitophagy, and therefore its specific contribution remains incompletely defined. Deletion of *PINK1* in skeletal muscle reduces LC3-II flux in mitochondria-enriched skeletal muscle following endurance exercise, suggesting that Parkin may be required for exercise-induced autophagy; however, these studies did not directly assess mitophagy [59]. Similarly, studies using mice expressing the fluorescent reporter gene *pMitotimer* and subjected to acute treadmill showed that PINK1 did not accumulate in the mitochondria-enriched fractions, indicating that PINK1 is dispensable for exercise-induced mitophagy [60]. Despite these advances, information regarding the molecular drivers of mitochondrial fragmentation and mechanisms regulating mitochondrial quality in skeletal muscle during HF is unknown. Furthermore, no study has been able to clearly demonstrate that PINK1-Parkin-mediated mitochondrial ubiquitylation occurs in skeletal muscle either in normal or HF conditions. Additionally, the sex differences in mitochondrial dynamics of HF patients warrant further investigation to develop a personalized therapeutic strategy.

### 4.4. Defects in Oxidative Phosphorylation (OXPHOS)

OXPHOS is the primary mechanism of ATP generation. It is comprised of five inner mitochondrial–protein complexes: complex I, complex II, dimeric complex III, complex IV, and complex V. And it is comprised of two electron carriers: membrane-embedded hydrophobic ubiquinone and soluble cytochrome c. Together, these form an electron transport chain (ETC). Complexes I, III, and IV, function as proton pumps. Complex II reduces ubiquinone and serves as electron transporter for complex III, which reduces cytochrome c that, in turn, shuttles to complex IV and donates its electron for the final reduction of oxygen. The generated proton is used by the ATP synthase, which phosphorylates ADP to ATP.

Previous work assessing mitochondrial respiratory rates in skeletal muscle biopsies of HF patients and correlating these with whole-body exercise capacity using cardiopulmonary exercise test (CPET) has shown that mitochondrial respiration (complex I, II, and IV) as well as electron transport chain (ETC) capacity correlates strongly with VO_2 peak_, lactate threshold (VO_2LT_), and peak circulatory power CirP_peak_. Importantly, the mitochondrial ETC capacity contributes around 50% of the variability in VO_2LT_ peak, a key prognostic marker of exercise limitation in HF [61].

**Figure 2:**
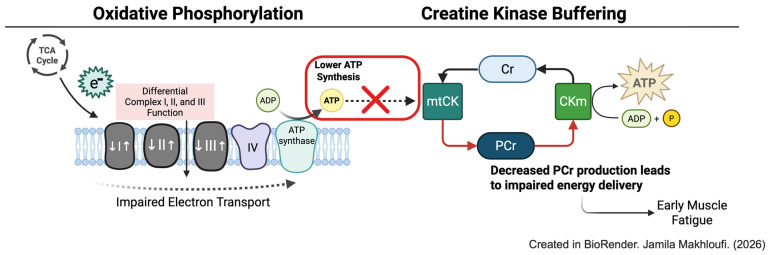
Mitochondrial dysfunction and impaired creatine kinase-mediated energy transfer in skeletal muscle during heart failure (HF). Mitochondria in the skeletal muscle undergoes biphasic responses during HF. State I, II, and III respiration rates are increased (↑) and decreased (↓) during compensated hypertrophy and onset of heart failure respectively, impairing electron transport and ATP synthesis and diminished creatine kinase buffering capacity.

Numerous studies have reported OXPHOS dysregulation in skeletal muscle during the development of HF. In rats subjected to pressure overload-induced HF, a biphasic response of mitochondrial function was observed during the transition from compensated hypertrophy to heart failure [62]. During the first 6 weeks of compensated hypertrophy, state 3 respiration in the gastrocnemius and soleus increases, followed by a decline between 10 and 20 weeks of TAC. Notably, the ADP/O ratios remained unchanged during the transition from hypertrophy to heart failure. Similarly, activities of complex I and II activities are elevated in the gastrocnemius and soleus muscles during the compensated stage of cardiac remodeling but decrease substantially with the onset of HF (Figure 2). These changes in the activities of these complexes have been attributed to either changes in the mRNA and protein expression or impaired assembly of respiratory complex subunits to super complex. In human HF patients, decreases in complex activities are accompanied by a decrease in mRNA and protein expressions of complex I, complex II, and complex III [63,64]. Studies in pacing-induced HF in dogs further reveal that impaired oxidative phosphorylation is not only restricted to defects localized in the ETC but also in the components of the phosphorylation system, such as reduced ANT2 and increased ANT1 expression [65].

## 5. Phosphocreatine Depletion

The ATP generated by oxidative phosphorylation is transferred to mitochondrial creatine kinase (mi-CK), present on the outer surface of the inner mitochondrial membrane. mi-CK transfers the phosphate moiety of ATP to creatine, producing phosphocreatine (PCr) [66]. PCr plays a critical role as a rapid high-energy phosphate reservoir in skeletal muscle through CK, enabling immediate ATP production during periods of high-energy demand [67]. It is also crucial for cellular energy homeostasis during muscle contraction. PCr serves as an energy carrier for the connecting sites of energy production with energy utilization via subcellular compartmentalization. Several studies in both human subjects and animal models of HF have reported significantly reduced storage of PCr, accompanied by faster PCr depletion and slower recovery in the skeletal muscle under exercise conditions [68,69,70]. Muscle tissue from heart failure mice and humans showed mi-CK activity had a dramatic decrease, of approximately 60–80% compared with the controls (Figure 2) [71,72]. Nagai et al. further reported there is a significant correlation between the PCr breakdown and peakV(O)_2_ in both the arms and legs, as well as a close relationship between the ventilatory anaerobic threshold and the PCr breakdown [28]. These findings suggest that impaired PCr metabolism is closely linked to exercise intolerance in HF patients.

## 6. Oxidative Stress and Lipid Peroxidation in the Skeletal Muscle

Oxidative stress arises from an imbalance between oxidant production and antioxidant defenses, leading to excessive generation of reactive oxygen species (ROS), including superoxide anion (O_2_^−^), hydroxyl radical (•OH), and hydrogen peroxide (H_2_O_2_). ROS are generated through several processes, such as the mitochondrial respiratory chain, NAD(P)H oxidase, and xanthine oxidases (XO). In addition, impaired antioxidant capacity, resulting from decreased expression of antioxidant enzymes, further exacerbates oxidative stress.

Extensive clinical and experimental evidence shows that HF is associated with increased oxidative stress in skeletal muscle. In patients with HF, inducible NOS (iNOS) expression is elevated in skeletal muscle and inversely correlates with maximal oxygen uptake and exercise tolerance [73,74]. Moreover, xanthine oxidase (XO), which converts xanthine to uric acid while generating superoxide and hydrogen peroxide, is activated in the slow-twitch muscle fibers (type I and IIa) during the acute phase of myocardial infarction in mice [36]. This activation contributes to mitochondrial dysfunction, reduced exercise capacity, and apoptosis. Notably, pharmacological inhibition of XO with febuxostat prevents exercise intolerance without affecting left ventricular function or remodeling [36]. Studies in animal models of HF further show that overexpression of superoxide dismutase in the skeletal muscle or antioxidant supplementation improves muscle function [75,76,77]. However, clinical trials of antioxidants therapies have largely failed to demonstrate benefit in HF patients [77].

**Figure 3:**
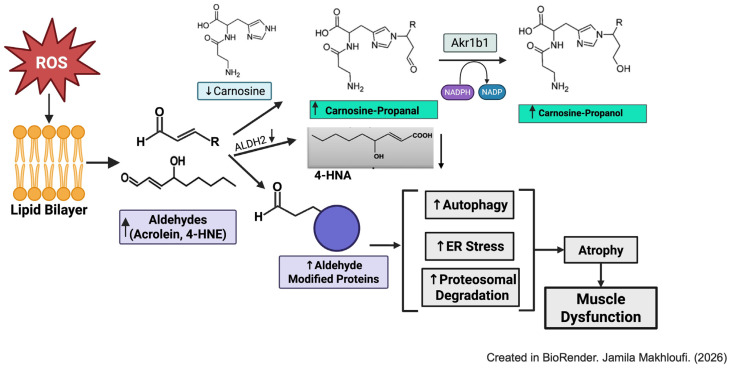
Oxidative stress, lipid peroxidation formation, and aldehyde detoxification is impaired in the skeletal muscle during heart failure. Reactive oxygen species (ROS) and toxic lipid peroxidation products, including 4-hydroxynonenal (4-HNE) and acrolein, are accumulated in the skeletal muscle during heart failure. Aldehyde detoxification pathways, such as aldehyde dehydrogenase 2 (ALDH2) and carnosine are decreased (↓) in the skeletal muscle during heart failure, leading to decreased formation of 4-hydroxynonenic acid (4-HNA), (↑) accumulation of carnosine-propanal and carnosine propanol. (↑) Accumulation of aldehydes increases the formation of aldehyde modified protein adducts, activating autophagy, ER stress, and proteasomal degradation in the muscle.

Excessive ROS generation induces the formation of highly reactive lipid peroxidation products, such as acrolein, 4-hydroxnonenal (4-HNE), and malondialdehyde, produced through the oxidation of polyunsaturated fatty acids (PUFA) in cellular membranes. These aldehydes possess two reactive carbonyl groups that form covalent adducts with amino acid side chains and nucleophilic sites of DNA, forming aldehyde-modified proteins and DNA adducts [78,79]. Lipid-derived aldehydes activate catabolic pathways including the ubiquitin proteosome pathway (UPS), autophagy, and inflammation, leading to muscle degradation [78,80]. They also impair membrane integrity, disrupt mitochondrial function and ATP production, promote apoptosis, and contribute to sarcopenia [81]. Detoxification of these lipid peroxidation products occurs primarily via enzymatic reduction by aldehyde dehydrogenase (ALDH2) and aldose reductase (Akr1b1) [82]. Additionally, aldehydes are scavenged through conjugation with the nucleophile glutathione [83] and endogenous histidyl dipeptides such as carnosine (β-alanine-histidine) and anserine (β-alanine-N^π^-histidine), as shown in Figure 3 [84,85]. To determine whether these toxic aldehydes accumulate in the skeletal muscle during HF, we recently showed that mice subjected to transaortic constriction for 12 weeks exhibit significantly elevated levels of acrolein and 4-HNE protein adducts in the skeletal muscle, accompanied by reduced skeletal muscle weight, strength, and decreased ALDH2 expression. Importantly, both carnosine levels and the expression of enzyme carnosine synthase, the enzyme which synthesizes carnosine, were reduced in the skeletal muscles of HF mice [80]. These findings suggest that skeletal muscle carnosine may serve as a diagnostic marker of skeletal muscle dysfunction during HF.

## 7. Therapeutic Interventions Targeting Skeletal Muscle in Heart Failure

Exercise training is an evidence-based intervention that reverses skeletal muscle dysfunction in HF. Previous studies in patients with HF have shown that exercise training reduces inflammatory cytokine levels in skeletal muscle and improves muscle function [86,87,88]. However, due to limited efficacy and durability, exercise cannot be a standalone intervention in these patients.

Emerging pharmacological therapies aim to address the metabolic and molecular derangements observed in HF skeletal muscle. Mitochondria-targeted antioxidants, ACE inhibitors, calcium sensitizers, and metabolic modulators have been tested and continue to be explored to restore energy homeostasis and improve skeletal performance in HF patients. A recent report in heart failure mice showed that targeting mitochondrial redox signaling by modulating the SIRT3–SOD2 axis in the skeletal muscle improves exercise tolerance. Treatment with Honokiol, a SIRT3 activator, resulted in SOD2 deacetylation and activation reduced mitochondrial ROS, restored oxidative capacity, and significantly improved exercise performance. Furthermore, skeletal muscle-targeted SIRT3 overexpression via AAV9 improved exercise capacity, confirming that the modulation of mitochondrial quality in muscle alone is sufficient to enhance functional performance in heart failure [89]. Mitochondrial ROS are a primary contributor of oxidative stress in skeletal muscle during HF. Elamipretide, a mitochondria targeted tetrapeptide (also known as MTP-131, SS-31), crosses the plasma membrane, localizes to the inner mitochondrial membrane, binds with cardiolipin, and enhances the flux of electrons through cytochrome c. In addition, elamipretide reduces mitochondrial ROS production [90]. Treatment with elamipretide has been shown to improve mitochondrial respiratory function and restore ATP synthesis and the ATP/ADP ratio in the skeletal muscle of dogs with experimentally induced HF [91]. While elamipretide is safe and tolerated in HF patients [92,93], its impact on exercise performance has not been directly tested.

ACE inhibitors are an established intervention for chronic heart failure (CHF) that also improve skeletal muscle dysfunction in both human and animal model of HF. In HF patients, ACE inhibition increases the proportion of slow oxidative MHCI fibers and decreases fast glycolytic MHCII, changes that correlate with improved exercise tolerance [94]. Additionally, several studies have shown that testosterone supplementation improves exercise capacity, muscle strength, and glucose metabolism in HF. In a pilot study of CHF patients, intramuscular testosterone therapy improved the distance covered in a shuttle walking test [95]. Similarly, a placebo-controlled trial demonstrated that 12 months of testosterone replacement therapy in men with moderately severe CHF improved the distance covered on shuttle walking and forearm strength [96]. Short-term administration of testosterone in HF patients also improved the baseline peak oxygen consumption, ventilatory efficiency, and isometric quadricep strength [97]. Although the mechanisms by which testosterone improves muscle strength in these patients remain unclear, studies in healthy mice show that administration of testosterone increases both the number and size of type I slow oxidative fibers [98], suggesting that testosterone may improve the oxidative capacity in HF skeletal muscle.

Studies using SGLT2 inhibitors in both murine models and human HF indicate that improvements in exercise performance may result from beneficial effects on skeletal muscle pathology. In mice subjected to myocardial infarction and subsequently treated with SGLT2 inhibitor empagliflozin, improved exercise capacity was linked to increased fatty acid oxidation and oxidative phosphorylation in the skeletal muscle [99]. Similarly, HF patients treated with SGLT2 inhibitor dapagliflozin improved exercise capacity, correlated with increased tryptophan metabolism and enrichment of anti-atrophic transcriptomic profile in skeletal muscle [100].

Levosimendan, a calcium sensitizer used clinically for HF, improves forelimb grip strength and distance and time running time in mice with HF. Treatment increased the cross-sectional area of skeletal muscle fibers, mitochondrial content, and mitochondrial membrane potential, and the improvement in exercise performance was independent of changes in cardiac function [101]. Similarly, a recent study in patients with HF showed that intravenous injection of iron in iron-deficient patients improves skeletal muscle function by enhancing energy metabolism and reducing reliance on glycolytic ATP production [102].

Molecular interventions targeting muscle regeneration pathways and histidyl dipeptide metabolism also hold promise. Carnosine, an endogenous histidyl dipeptide with aldehyde-scavenging properties, is depleted in HF skeletal muscle [80]. Supplementation strategies using β-alanine or carnosine precursors may restore muscle detoxification capacity, reduce aldehyde-induced protein damage, and improve exercise performance.

## 8. Conclusions

Skeletal muscle dysfunction is a critical contributor to exercise intolerance in HF. Disruptions in fiber type composition, mitochondrial structure and function, oxidative phosphorylation, and insulin signaling, compounded by oxidative stress and aldehyde accumulation, create a self-perpetuating cycle of metabolic impairment, fatigue, and atrophy. Emerging pharmacological, molecular, and nutritional strategies targeting mitochondrial health, oxidative stress, and histidyl dipeptide metabolism represent promising approaches to enhance skeletal muscle performance and exercise tolerance in HF patients. Future research should be focused on elucidating molecular mechanisms driving skeletal muscle dysfunction, including sex-specific differences in mitochondrial dynamics and regulation; evaluating therapeutic strategies that restore metabolic flexibility and improve mitochondrial quality; and investigating combinatorial approaches integrating exercise, pharmacological agents, and molecular interventions to maximize functional recovery in HF skeletal muscle. Addressing these gaps is essential to developing therapies that preserve muscle function, improve exercise tolerance, and ultimately enhance quality of life and outcomes for HF patients.

## Data Availability

No new data were created or analyzed in this study. Data sharing is not applicable to this article.

## Abbreviations

The following abbreviations are used in this manuscript:

ATAD3	domain-containing protein 3
Ca^2+^	calcium
CFR	coronary flow reserve
CK	creatine kinase
CHF	chronic heart failure
ETC	electron transport chain
MHC	myosin heavy chain
(mi-CK)	mitochondrial creatine kinase
mtDNA	mitochondrial DNA
GLUT4	primary-insulin regulated glucose transporter protein
HF	heart failure
4-HNE	4-Hydroxynonenal
i-NOS	inducible nitric oxide synthase
OXPHOS	oxidative phosphorylation
PCr	phosphocreatine
PINK1	PTEN-induced putative kinase
POLG	DNA polymerase gamma, catalytic subunit
ROS	reactive oxygen species
TAC	transaortic constriction
TFAM	mitochondrial transcription factor A
Tn	troponin
Tm	tropomyosin
XO	xanthine oxidase

## References

[1] Maskin C.S., Forman R., Sonnenblick E.H., Frishman W.H., LeJemtel T.H. (1983). Failure of dobutamine to increase exercise capacity despite hemodynamic improvement in severe chronic heart failure. Am. J. Cardiol..

[2] Wilson J.R., Martin J.L., Ferraro N. (1984). Impaired skeletal muscle nutritive flow during exercise in patients with congestive heart failure: Role of cardiac pump dysfunction as determined by the effect of dobutamine. Am. J. Cardiol..

[3] Wiener D.H., Fink L.I., Maris J., Jones R.A., Chance B., Wilson J.R. (1986). Abnormal skeletal muscle bioenergetics during exercise in patients with heart failure: Role of reduced muscle blood flow. Circulation.

[4] Richard L.L. (2010). Skeletal Muscle Structure, Function, and Plasticity.

[5] van Hees H.W., van der Heijden H.F., Ottenheijm C.A., Heunks L.M., Pigmans C.J., Verheugt F.W., Brouwer R.M., Dekhuijzen P.N. (2007). Diaphragm single-fiber weakness and loss of myosin in congestive heart failure rats. Am. J. Physiol. Heart Circ. Physiol..

[6] Miller M.S., Vanburen P., Lewinter M.M., Lecker S.H., Selby D.E., Palmer B.M., Maughan D.W., Ades P.A., Toth M.J. (2009). Mechanisms underlying skeletal muscle weakness in human heart failure: Alterations in single fiber myosin protein content and function. Circ. Heart Fail..

[7] Toth M.J., Matthews D.E., Ades P.A., Tischler M.D., Van Buren P., Previs M., LeWinter M.M. (2005). Skeletal muscle myofibrillar protein metabolism in heart failure: Relationship to immune activation and functional capacity. Am. J. Physiol. Endocrinol. Metab..

[8] Coirault C., Guellich A., Barbry T., Samuel J.L., Riou B., Lecarpentier Y. (2007). Oxidative stress of myosin contributes to skeletal muscle dysfunction in rats with chronic heart failure. Am. J. Physiol. Heart Circ. Physiol..

[9] Maher J.C. (1991). Differences in mortality from coronary artery bypass graft surgery. JAMA.

[10] Miller M.S., VanBuren P., LeWinter M.M., Braddock J.M., Ades P.A., Maughan D.W., Palmer B.M., Toth M.J. (2010). Chronic heart failure decreases cross-bridge kinetics in single skeletal muscle fibres from humans. J. Physiol..

[11] Spangenburg E.E., Talmadge R.J., Musch T.I., Pfeifer P.C., McAllister R.M., Williams J.H. (2002). Changes in skeletal muscle myosin heavy chain isoform content during congestive heart failure. Eur. J. Appl. Physiol..

[12] Sabbah H.N., Hansen-Smith F., Sharov V.G., Kono T., Lesch M., Gengo P.J., Steffen R.P., Levine T.B., Goldstein S. (1993). Decreased proportion of type I myofibers in skeletal muscle of dogs with chronic heart failure. Circulation.

[13] Mancini D.M., Coyle E., Coggan A., Beltz J., Ferraro N., Montain S., Wilson J.R. (1989). Contribution of intrinsic skeletal muscle changes to 31P NMR skeletal muscle metabolic abnormalities in patients with chronic heart failure. Circulation.

[14] Dunnigan A., Staley N.A., Smith S.A., Pierpont M.E., Judd D., Benditt D.G., Benson D.W. (1987). Cardiac and skeletal muscle abnormalities in cardiomyopathy: Comparison of patients with ventricular tachycardia or congestive heart failure. J. Am. Coll. Cardiol..

[15] Lipkin D.P., Jones D.A., Round J.M., Poole-Wilson P.A. (1988). Abnormalities of skeletal muscle in patients with chronic heart failure. Int. J. Cardiol..

[16] Vescovo G., Serafini F., Facchin L., Tenderini P., Carraro U., Dalla Libera L., Catani C., Ambrosio G.B. (1996). Specific changes in skeletal muscle myosin heavy chain composition in cardiac failure: Differences compared with disuse atrophy as assessed on microbiopsies by high resolution electrophoresis. Heart.

[17] Vescovo G., Serafini F., Dalla Libera L., Leprotti C., Facchin L., Tenderini P., Ambrosio G.B. (1998). Skeletal muscle myosin heavy chains in heart failure: Correlation between magnitude of the isozyme shift, exercise capacity, and gas exchange measurements. Am. Heart J..

[18] Sullivan M.J., Green H.J., Cobb F.R. (1990). Skeletal muscle biochemistry and histology in ambulatory patients with long-term heart failure. Circulation.

[19] Doehner W., Turhan G., Leyva F., Rauchhaus M., Sandek A., Jankowska E.A., von Haehling S., Anker S.D. (2015). Skeletal muscle weakness is related to insulin resistance in patients with chronic heart failure. ESC Heart Fail..

[20] Doehner W., Rauchhaus M., Godsland I.F., Egerer K., Niebauer J., Sharma R., Cicoira M., Florea V.G., Coats A.J., Anker S.D. (2002). Insulin resistance in moderate chronic heart failure is related to hyperleptinaemia, but not to norepinephrine or TNF-alpha. Int. J. Cardiol..

[21] Doehner W., Gathercole D., Cicoira M., Krack A., Coats A.J., Camici P.G., Anker S.D. (2010). Reduced glucose transporter GLUT4 in skeletal muscle predicts insulin resistance in non-diabetic chronic heart failure patients independently of body composition. Int. J. Cardiol..

[22] Kemppainen J., Tsuchida H., Stolen K., Karlsson H., Bjornholm M., Heinonen O.J., Nuutila P., Krook A., Knuuti J., Zierath J.R. (2003). Insulin signalling and resistance in patients with chronic heart failure. J. Physiol..

[23] Ohta Y., Kinugawa S., Matsushima S., Ono T., Sobirin M.A., Inoue N., Yokota T., Hirabayashi K., Tsutsui H. (2011). Oxidative stress impairs insulin signal in skeletal muscle and causes insulin resistance in postinfarct heart failure. Am. J. Physiol. Heart Circ. Physiol..

[24] Velasquez F.C., Roman B., Hernandez-Ochoa E.O., Leppo M.K., Truong S.K., Steenbergen C., Schneider M.F., Weiss R.G., Das S. (2023). Contribution of skeletal muscle-specific microRNA-133b to insulin resistance in heart failure. Am. J. Physiol. Heart Circ. Physiol..

[25] Snoer M., Monk-Hansen T., Olsen R.H., Pedersen L.R., Simonsen L., Rasmusen H., Dela F., Prescott E. (2012). Insulin resistance and exercise tolerance in heart failure patients: Linkage to coronary flow reserve and peripheral vascular function. Cardiovasc. Diabetol..

[26] Santos J.M., Kowatsch I., Tsutsui J.M., Negrao C.E., Canavesi N., Carvalho Frimm C., Mady C., Ramires J.A., Mathias W. (2010). Effects of exercise training on myocardial blood flow reserve in patients with heart failure and left ventricular systolic dysfunction. Am. J. Cardiol..

[27] Kelley D., Mitrakou A., Marsh H., Schwenk F., Benn J., Sonnenberg G., Arcangeli M., Aoki T., Sorensen J., Berger M. (1988). Skeletal muscle glycolysis, oxidation, and storage of an oral glucose load. J. Clin. Investig..

[28] Nagai T., Okita K., Yonezawa K., Yamada Y., Hanada A., Ohtsubo M., Morita N., Murakami T., Nishijima H., Kitabatake A. (2004). Comparisons of the skeletal muscle metabolic abnormalities in the arm and leg muscles of patients with chronic heart failure. Circ. J..

[29] Clark A.L., Poole-Wilson P.A., Coats A.J. (1996). Exercise limitation in chronic heart failure: Central role of the periphery. J. Am. Coll. Cardiol..

[30] Mancini D.M., Ferraro N., Tuchler M., Chance B., Wilson J.R. (1988). Detection of abnormal calf muscle metabolism in patients with heart failure using phosphorus-31 nuclear magnetic resonance. Am. J. Cardiol..

[31] Massie B.M., Conway M., Yonge R., Frostick S., Sleight P., Ledingham J., Radda G., Rajagopalan B. (1987). 31P nuclear magnetic resonance evidence of abnormal skeletal muscle metabolism in patients with congestive heart failure. Am. J. Cardiol..

[32] Schaufelberger M., Eriksson B.O., Grimby G., Held P., Swedberg K. (1997). Skeletal muscle alterations in patients with chronic heart failure. Eur. Heart J..

[33] Caspi T., Straw S., Cheng C., Garnham J.O., Scragg J.L., Smith J., Koshy A.O., Levelt E., Sukumar P., Gierula J. (2020). Unique Transcriptome Signature Distinguishes Patients with Heart Failure with Myopathy. J. Am. Heart Assoc..

[34] Drexler H., Riede U., Munzel T., Konig H., Funke E., Just H. (1992). Alterations of skeletal muscle in chronic heart failure. Circulation.

[35] Guzman Mentesana G., Baez A.L., Lo Presti M.S., Dominguez R., Cordoba R., Bazan C., Strauss M., Fretes R., Rivarola H.W., Paglini-Oliva P. (2014). Functional and structural alterations of cardiac and skeletal muscle mitochondria in heart failure patients. Arch. Med. Res..

[36] Nambu H., Takada S., Maekawa S., Matsumoto J., Kakutani N., Furihata T., Shirakawa R., Katayama T., Nakajima T., Yamanashi K. (2021). Inhibition of xanthine oxidase in the acute phase of myocardial infarction prevents skeletal muscle abnormalities and exercise intolerance. Cardiovasc. Res..

[37] Tabony A.M., Yoshida T., Sukhanov S., Delafontaine P. (2014). Protein phosphatase 2C-alpha knockdown reduces angiotensin II-mediated skeletal muscle wasting via restoration of mitochondrial recycling and function. Skelet. Muscle.

[38] Zoll J., Monassier L., Garnier A., N’Guessan B., Mettauer B., Veksler V., Piquard F., Ventura-Clapier R., Geny B. (2006). ACE inhibition prevents myocardial infarction-induced skeletal muscle mitochondrial dysfunction. J. Appl. Physiol..

[39] Toth M.J., Miller M.S., Ward K.A., Ades P.A. (2012). Skeletal muscle mitochondrial density, gene expression, and enzyme activities in human heart failure: Minimal effects of the disease and resistance training. J. Appl. Physiol..

[40] Wredenberg A., Wibom R., Wilhelmsson H., Graff C., Wiener H.H., Burden S.J., Oldfors A., Westerblad H., Larsson N.G. (2002). Increased mitochondrial mass in mitochondrial myopathy mice. Proc. Natl. Acad. Sci. USA.

[41] Theilen N.T., Jeremic N., Weber G.J., Tyagi S.C. (2019). TFAM overexpression diminishes skeletal muscle atrophy after hindlimb suspension in mice. Arch. Biochem. Biophys..

[42] Kawakami R., Matsui H., Matsui M., Iso T., Yokoyama T., Ishii H., Kurabayashi M. (2023). Empagliflozin induces the transcriptional program for nutrient homeostasis in skeletal muscle in normal mice. Sci. Rep..

[43] Santos-Gallego C.G., Vargas-Delgado A.P., Requena-Ibanez J.A., Garcia-Ropero A., Mancini D., Pinney S., Macaluso F., Sartori S., Roque M., Sabatel-Perez F. (2021). Randomized Trial of Empagliflozin in Nondiabetic Patients With Heart Failure and Reduced Ejection Fraction. J. Am. Coll. Cardiol..

[44] Palau P., Amiguet M., Dominguez E., Sastre C., Mollar A., Seller J., Garcia Pinilla J.M., Larumbe A., Valle A., Gomez Doblas J.J. (2022). Short-term effects of dapagliflozin on maximal functional capacity in heart failure with reduced ejection fraction (DAPA-VO(2)): A randomized clinical trial. Eur. J. Heart Fail..

[45] Sesaki H., Southard S.M., Yaffe M.P., Jensen R.E. (2003). Mgm1p, a dynamin-related GTPase, is essential for fusion of the mitochondrial outer membrane. Mol. Biol. Cell.

[46] Hales K.G., Fuller M.T. (1997). Developmentally regulated mitochondrial fusion mediated by a conserved, novel, predicted GTPase. Cell.

[47] Tilokani L., Nagashima S., Paupe V., Prudent J. (2018). Mitochondrial dynamics: Overview of molecular mechanisms. Essays Biochem..

[48] Chan D.C. (2006). Mitochondrial fusion and fission in mammals. Annu. Rev. Cell Dev. Biol..

[49] Smirnova E., Griparic L., Shurland D.L., van der Bliek A.M. (2001). Dynamin-related protein Drp1 is required for mitochondrial division in mammalian cells. Mol. Biol. Cell.

[50] Chan D.C. (2012). Fusion and fission: Interlinked processes critical for mitochondrial health. Annu. Rev. Genet..

[51] Narendra D., Tanaka A., Suen D.F., Youle R.J. (2008). Parkin is recruited selectively to impaired mitochondria and promotes their autophagy. J. Cell Biol..

[52] Geisler S., Holmstrom K.M., Skujat D., Fiesel F.C., Rothfuss O.C., Kahle P.J., Springer W. (2010). PINK1/Parkin-mediated mitophagy is dependent on VDAC1 and p62/SQSTM1. Nat. Cell Biol..

[53] Kawajiri S., Saiki S., Sato S., Sato F., Hatano T., Eguchi H., Hattori N. (2010). PINK1 is recruited to mitochondria with parkin and associates with LC3 in mitophagy. FEBS Lett..

[54] Matsuda N., Sato S., Shiba K., Okatsu K., Saisho K., Gautier C.A., Sou Y.S., Saiki S., Kawajiri S., Sato F. (2010). PINK1 stabilized by mitochondrial depolarization recruits Parkin to damaged mitochondria and activates latent Parkin for mitophagy. J. Cell Biol..

[55] Vives-Bauza C., Zhou C., Huang Y., Cui M., de Vries R.L., Kim J., May J., Tocilescu M.A., Liu W., Ko H.S. (2010). PINK1-dependent recruitment of Parkin to mitochondria in mitophagy. Proc. Natl. Acad. Sci. USA.

[56] Gortan Cappellari G., Aleksova A., Dal Ferro M., Cannata A., Semolic A., Guarnaccia A., Zanetti M., Giacca M., Sinagra G., Barazzoni R. (2023). n-3 PUFA-Enriched Diet Preserves Skeletal Muscle Mitochondrial Function and Redox State and Prevents Muscle Mass Loss in Mice with Chronic Heart Failure. Nutrients.

[57] Rodriguez J., Specian V., Maloney R., Jourd’heuil D., Feelisch M. (2005). Performance of diamino fluorophores for the localization of sources and targets of nitric oxide. Free Radic. Biol. Med..

[58] Garnham J.O., Roberts L.D., Caspi T., Al-Owais M.M., Bullock M., Swoboda P.P., Koshy A., Gierula J., Paton M.F., Cubbon R.M. (2020). Divergent skeletal muscle mitochondrial phenotype between male and female patients with chronic heart failure. J. Cachexia Sarcopenia Muscle.

[59] Chen Z., Liu L., Cheng Q., Li Y., Wu H., Zhang W., Wang Y., Sehgal S.A., Siraj S., Wang X. (2017). Mitochondrial E3 ligase MARCH5 regulates FUNDC1 to fine-tune hypoxic mitophagy. EMBO Rep..

[60] Drake J.C., Laker R.C., Wilson R.J., Zhang M., Yan Z. (2019). Exercise-induced mitophagy in skeletal muscle occurs in the absence of stabilization of Pink1 on mitochondria. Cell Cycle.

[61] Knuiman P., Straw S., Gierula J., Koshy A., Roberts L.D., Witte K.K., Ferguson C., Bowen T.S. (2021). Quantifying the relationship and contribution of mitochondrial respiration to systemic exercise limitation in heart failure. ESC Heart Fail..

[62] Schrepper A., Schwarzer M., Schope M., Amorim P.A., Doenst T. (2012). Biphasic response of skeletal muscle mitochondria to chronic cardiac pressure overload—Role of respiratory chain complex activity. J. Mol. Cell Cardiol..

[63] Garnier A., Fortin D., Delomenie C., Momken I., Veksler V., Ventura-Clapier R. (2003). Depressed mitochondrial transcription factors and oxidative capacity in rat failing cardiac and skeletal muscles. J. Physiol..

[64] Rosca M.G., Vazquez E.J., Kerner J., Parland W., Chandler M.P., Stanley W., Sabbah H.N., Hoppel C.L. (2008). Cardiac mitochondria in heart failure: Decrease in respirasomes and oxidative phosphorylation. Cardiovasc. Res..

[65] Rosca M.G., Okere I.A., Sharma N., Stanley W.C., Recchia F.A., Hoppel C.L. (2009). Altered expression of the adenine nucleotide translocase isoforms and decreased ATP synthase activity in skeletal muscle mitochondria in heart failure. J. Mol. Cell Cardiol..

[66] Saks V., Favier R., Guzun R., Schlattner U., Wallimann T. (2006). Molecular system bioenergetics: Regulation of substrate supply in response to heart energy demands. J. Physiol..

[67] Menuet C., Arsac L.M. (2008). Muscle [phosphocreatine] dynamics during exercise: Implication for understanding the regulation of muscle oxidative metabolism. J. Physiol..

[68] Bernocchi P., Cargnoni A., Vescovo G., Dalla Libera L., Parrinello G., Boraso A., Ceconi C., Ferrari R. (2003). Skeletal muscle abnormalities in rats with experimentally induced heart hypertrophy and failure. Basic. Res. Cardiol..

[69] van der Ent M., Jeneson J.A., Remme W.J., Berger R., Ciampricotti R., Visser F. (1998). A non-invasive selective assessment of type I fibre mitochondrial function using 31P NMR spectroscopy. Evidence for impaired oxidative phosphorylation rate in skeletal muscle in patients with chronic heart failure. Eur. Heart J..

[70] Mancini D.M., Walter G., Reichek N., Lenkinski R., McCully K.K., Mullen J.L., Wilson J.R. (1992). Contribution of skeletal muscle atrophy to exercise intolerance and altered muscle metabolism in heart failure. Circulation.

[71] De Sousa E., Veksler V., Minajeva A., Kaasik A., Mateo P., Mayoux E., Hoerter J., Bigard X., Serrurier B., Ventura-Clapier R. (1999). Subcellular creatine kinase alterations. Implications in heart failure. Circ. Res..

[72] Mettauer B., Zoll J., Sanchez H., Lampert E., Ribera F., Veksler V., Bigard X., Mateo P., Epailly E., Lonsdorfer J. (2001). Oxidative capacity of skeletal muscle in heart failure patients versus sedentary or active control subjects. J. Am. Coll. Cardiol..

[73] Adams V., Yu J., Mobius-Winkler S., Linke A., Weigl C., Hilbrich L., Schuler G., Hambrecht R. (1997). Increased inducible nitric oxide synthase in skeletal muscle biopsies from patients with chronic heart failure. Biochem. Mol. Med..

[74] Hambrecht R., Adams V., Gielen S., Linke A., Mobius-Winkler S., Yu J., Niebauer J., Jiang H., Fiehn E., Schuler G. (1999). Exercise intolerance in patients with chronic heart failure and increased expression of inducible nitric oxide synthase in the skeletal muscle. J. Am. Coll. Cardiol..

[75] Okutsu M., Call J.A., Lira V.A., Zhang M., Donet J.A., French B.A., Martin K.S., Peirce-Cottler S.M., Rembold C.M., Annex B.H. (2014). Extracellular superoxide dismutase ameliorates skeletal muscle abnormalities, cachexia, and exercise intolerance in mice with congestive heart failure. Circ. Heart Fail..

[76] Hauer K., Hildebrandt W., Sehl Y., Edler L., Oster P., Droge W. (2003). Improvement in muscular performance and decrease in tumor necrosis factor level in old age after antioxidant treatment. J. Mol. Med..

[77] Mantovani G., Maccio A., Madeddu C., Gramignano G., Lusso M.R., Serpe R., Massa E., Astara G., Deiana L. (2006). A phase II study with antioxidants, both in the diet and supplemented, pharmaconutritional support, progestagen, and anti-cyclooxygenase-2 showing efficacy and safety in patients with cancer-related anorexia/cachexia and oxidative stress. Cancer Epidemiol. Biomark. Prev..

[78] Baba S.P., Zhang D., Singh M., Dassanayaka S., Xie Z., Jagatheesan G., Zhao J., Schmidtke V.K., Brittian K.R., Merchant M.L. (2018). Deficiency of aldose reductase exacerbates early pressure overload-induced cardiac dysfunction and autophagy in mice. J. Mol. Cell Cardiol..

[79] Liu X., Lovell M.A., Lynn B.C. (2006). Detection and quantification of endogenous cyclic DNA adducts derived from trans-4-hydroxy-2-nonenal in human brain tissue by isotope dilution capillary liquid chromatography nanoelectrospray tandem mass spectrometry. Chem. Res. Toxicol..

[80] Chaudhari M., Zelko I., Lorkiewicz P., Hoetker D., Nong Y., Doelling B., Brittian K., Bhatnagar A., Srivastava S., Baba S.P. (2024). Metabolic pathways for removing reactive aldehydes are diminished in the skeletal muscle during heart failure. Skelet. Muscle.

[81] Eshima H., Shahtout J.L., Siripoksup P., Pearson M.J., Mahmassani Z.S., Ferrara P.J., Lyons A.W., Maschek J.A., Peterlin A.D., Verkerke A.R.P. (2023). Lipid hydroperoxides promote sarcopenia through carbonyl stress. eLife.

[82] Baba S.P., Hoetker J.D., Merchant M., Klein J.B., Cai J., Barski O.A., Conklin D.J., Bhatnagar A. (2013). Role of aldose reductase in the metabolism and detoxification of carnosine-acrolein conjugates. J. Biol. Chem..

[83] Srivastava S., Watowich S.J., Petrash J.M., Srivastava S.K., Bhatnagar A. (1999). Structural and kinetic determinants of aldehyde reduction by aldose reductase. Biochemistry.

[84] Blancquaert L., Baba S.P., Kwiatkowski S., Stautemas J., Stegen S., Barbaresi S., Chung W., Boakye A.A., Hoetker J.D., Bhatnagar A. (2016). Carnosine and anserine homeostasis in skeletal muscle and heart is controlled by beta-alanine transamination. J. Physiol..

[85] Zhao J., Conklin D.J., Guo Y., Zhang X., Obal D., Guo L., Jagatheesan G., Katragadda K., He L., Yin X. (2020). Cardiospecific Overexpression of ATPGD1 (Carnosine Synthase) Increases Histidine Dipeptide Levels and Prevents Myocardial Ischemia Reperfusion Injury. J. Am. Heart Assoc..

[86] Lavine K.J., Sierra O.L. (2017). Skeletal muscle inflammation and atrophy in heart failure. Heart Fail. Rev..

[87] Mann D.L., Reid M.B. (2003). Exercise training and skeletal muscle inflammation in chronic heart failure: Feeling better about fatigue. J. Am. Coll. Cardiol..

[88] Gielen S., Adams V., Mobius-Winkler S., Linke A., Erbs S., Yu J., Kempf W., Schubert A., Schuler G., Hambrecht R. (2003). Anti-inflammatory effects of exercise training in the skeletal muscle of patients with chronic heart failure. J. Am. Coll. Cardiol..

[89] Masunaga T., Suenaga T., Matsushima S., Hashimoto T., Takada S., Noda E., Fumoto Y., Hata S., Yokota T., Kinugawa S. (2025). Reduction in Acetylation of Superoxide Dismutase 2 in Skeletal Muscle Improves Exercise Capacity in Mice With Heart Failure. J. Cachexia Sarcopenia Muscle.

[90] Birk A.V., Liu S., Soong Y., Mills W., Singh P., Warren J.D., Seshan S.V., Pardee J.D., Szeto H.H. (2013). The mitochondrial-targeted compound SS-31 re-energizes ischemic mitochondria by interacting with cardiolipin. J. Am. Soc. Nephrol..

[91] Sabbah H.N., Gupta R.C., Singh-Gupta V., Zhang K. (2019). Effects of elamipretide on skeletal muscle in dogs with experimentally induced heart failure. ESC Heart Fail..

[92] Butler J., Khan M.S., Anker S.D., Fonarow G.C., Kim R.J., Nodari S., O’Connor C.M., Pieske B., Pieske-Kraigher E., Sabbah H.N. (2020). Effects of Elamipretide on Left Ventricular Function in Patients with Heart Failure with Reduced Ejection Fraction: The PROGRESS-HF Phase 2 Trial. J. Card. Fail..

[93] Daubert M.A., Yow E., Dunn G., Marchev S., Barnhart H., Douglas P.S., O’Connor C., Goldstein S., Udelson J.E., Sabbah H.N. (2017). Novel Mitochondria-Targeting Peptide in Heart Failure Treatment: A Randomized, Placebo-Controlled Trial of Elamipretide. Circ. Heart Fail..

[94] Vescovo G., Dalla Libera L., Serafini F., Leprotti C., Facchin L., Volterrani M., Ceconi C., Ambrosio G.B. (1998). Improved exercise tolerance after losartan and enalapril in heart failure: Correlation with changes in skeletal muscle myosin heavy chain composition. Circulation.

[95] Pugh P.J., Jones R.D., West J.N., Jones T.H., Channer K.S. (2004). Testosterone treatment for men with chronic heart failure. Heart.

[96] Malkin C.J., Pugh P.J., West J.N., van Beek E.J., Jones T.H., Channer K.S. (2006). Testosterone therapy in men with moderate severity heart failure: A double-blind randomized placebo controlled trial. Eur. Heart J..

[97] Caminiti G., Volterrani M., Iellamo F., Marazzi G., Massaro R., Miceli M., Mammi C., Piepoli M., Fini M., Rosano G.M. (2009). Effect of long-acting testosterone treatment on functional exercise capacity, skeletal muscle performance, insulin resistance, and baroreflex sensitivity in elderly patients with chronic heart failure a double-blind, placebo-controlled, randomized study. J. Am. Coll. Cardiol..

[98] Ustunel I., Akkoyunlu G., Demir R. (2003). The effect of testosterone on gastrocnemius muscle fibres in growing and adult male and female rats: A histochemical, morphometric and ultrastructural study. Anat. Histol. Embryol..

[99] Nambu H., Takada S., Fukushima A., Matsumoto J., Kakutani N., Maekawa S., Shirakawa R., Nakano I., Furihata T., Katayama T. (2020). Empagliflozin restores lowered exercise endurance capacity via the activation of skeletal muscle fatty acid oxidation in a murine model of heart failure. Eur. J. Pharmacol..

[100] Wood N., Straw S., Cheng C.W., Hirata Y., Pereira M.G., Gallagher H., Egginton S., Ogawa W., Wheatcroft S.B., Witte K.K. (2024). Sodium-glucose cotransporter 2 inhibitors influence skeletal muscle pathology in patients with heart failure and reduced ejection fraction. Eur. J. Heart Fail..

[101] Wang D., Song M., Shen L.F., Han L., Zhu P., Jia X., Shang G.K., Cao Y., Zhang W., Zhong M. (2021). Exercise Capacity Is Improved by Levosimendan in Heart Failure and Sarcopenia via Alleviation of Apoptosis of Skeletal Muscle. Front. Physiol..

[102] Drozd M.D., Tkaczyszyn M., Kasztura M., Wegrzynowska-Teodorczyk K., Flinta I., Banasiak W., Ponikowski P., Jankowska E.A. (2024). Intravenous iron supplementation improves energy metabolism of exercising skeletal muscles without effect on either oxidative stress or inflammation in male patients with heart failure with reduced ejection fraction. Cardiol. J..

